# Catabolism of Nucleic Acids by a Cystic Fibrosis *Pseudomonas aeruginosa* Isolate: An Adaptive Pathway to Cystic Fibrosis Sputum Environment

**DOI:** 10.3389/fmicb.2019.01199

**Published:** 2019-05-31

**Authors:** Sheemal Shanista Kumar, Anahit Penesyan, Liam Davin Hunt Elbourne, Michael R. Gillings, Ian T. Paulsen

**Affiliations:** ^1^Department of Molecular Sciences, Macquarie University, Sydney, NSW, Australia; ^2^Department of Biological Sciences, Macquarie University, Sydney, NSW, Australia

**Keywords:** *Pseudomonas aeruginosa*, cystic fibrosis, auxotrophy, carbon catabolism, biolog

## Abstract

*Pseudomonas aeruginosa* is a major cause of morbidity and mortality in patients with cystic fibrosis (CF). We undertook Biolog Phenotype Microarray testing of *P. aeruginosa* CF isolates to investigate their catabolic capabilities compared to *P. aeruginosa* laboratory strains PAO1 and PA14. One strain, PASS4, displayed an unusual phenotype, only showing strong respiration on adenosine and inosine. Further testing indicated that PASS4 could grow on DNA as a sole carbon source, with a higher biomass production than PAO1. This suggested that PASS4 was specifically adapted to metabolize extracellular DNA, a substrate present at high concentrations in the CF lung. Transcriptomic and proteomic profiling of PASS4 and PAO1 when grown with DNA as a sole carbon source identified a set of upregulated genes, including virulence and host-adaptation genes. PASS4 was unable to utilize *N*-Acetyl-D-glucosamine, and when we selected PASS4 mutants able to grow on this carbon source, they also displayed a gain in ability to catabolize a broad range of other carbon sources. Genome sequencing of the mutants revealed they all contained mutations within the *purK* gene, encoding a key protein in the *de novo* purine biosynthesis pathway. This suggested that PASS4 was a purine auxotroph. Growth assays in the presence of 2 mM adenosine and the complementation of PASS4 with an intact *purK* gene confirmed this conclusion. Purine auxotrophy may represent a viable microbial strategy for adaptation to DNA-rich environments such as the CF lung.

## Background

*Pseudomonas aeruginosa* is an opportunistic pathogen associated with complications in cystic fibrosis (CF) and is a major cause of death among CF patients. We found that *P. aeruginosa* CF isolate PASS4 has very limited carbon catabolism capabilities and may have become specialized at utilizing nucleic acids as a source of carbon. The CF lung contains viscous sputum which includes an abundance of DNA from epithelial and bacterial cells, and therefore provides an excellent niche for such an adaptation. We found that many *P. aeruginosa* virulence genes have increased expression in response to external DNA. Characterization of *P. aeruginosa* PASS4 revealed that the molecular basis of its metabolic specialization is a defect in biosynthesis of purines, precursors for DNA synthesis. Better understanding of *P. aeruginosa* adaptations in the CF lung will help in the development of specialized treatment regimes aimed at eradication of *P. aeruginosa* infections.

## Introduction

Cystic fibrosis (CF) is a genetic disorder most common among Caucasian populations (Petrova and Sauer, 2009). It is caused by mutations in the cystic fibrosis transmembrane regulator (*CFTR*) gene which encodes a cAMP-dependent chloride channel (Vankeerberghen et al., 2002). Dysfunction in the chloride channel leads to dehydrated and thickened airway surface liquid (ASL) hampering mucociliary clearance from the conducting airways (Mall, 2008). The thickened ASL enhances microbial colonization, leading to continuous stimulation of the immune system, and resulting in chronic lung inflammation (Sadikot et al., 2005). This hyperactive inflammatory response leads to a decline in lung function and eventual lung failure (Gellatly and Hancock, 2013).

*Pseudomonas aeruginosa* is one of the primary causes of acute and chronic lung infections in CF patients, resulting in significant morbidity and mortality (Wagner and Iglewski, 2008; Petrova and Sauer, 2009; Folkesson et al., 2012). *P. aeruginosa* is a metabolically versatile Gram-negative opportunistic pathogen that is common in various environments, such as soil and water (Wagner and Iglewski, 2008; Gellatly and Hancock, 2013). It can metabolize a broad range of carbon sources and grows both aerobically and anaerobically (Wagner and Iglewski, 2008; Mulcahy et al., 2010). Its metabolic versatility is conferred by a diverse set of transport systems and catabolic pathways encoded within a relatively large genome, typically more than 6 Mb (Winstanley et al., 2016).

Most CF patients who develop a lung infection by adolescence can live with the infection for 20 or more years (Gellatly and Hancock, 2013). During this period, *P. aeruginosa* continues to adapt to the CF lung environment. This results in the emergence of diverse phenotypes including traits such as increased mucoidy, auxotrophy, loss of motility, emergence of hypermutators, resistance to antimicrobials, and defects in key virulence factors such as quorum sensing regulation and type III secretion (Wagner and Iglewski, 2008; Folkesson et al., 2012; Gellatly and Hancock, 2013; Winstanley et al., 2016).

Adaptation and chronic or recurrent infection of *P. aeruginosa* in the CF lung is facilitated by its ability to grow as biofilms (Wagner and Iglewski, 2008). Biofilms are highly organized, structured bacterial communities attached to one another, and/or to an inert or living surface (Wagner and Iglewski, 2008; Petrova and Sauer, 2009; Gellatly and Hancock, 2013). Cells in biofilms are held together by a matrix of extracellular polymeric substances (EPS) consisting mainly of polysaccharides, extracellular DNA (eDNA), lipids, proteins, cellular debris, and membrane vesicles (Wagner and Iglewski, 2008; Gellatly and Hancock, 2013; Haussler and Fuqua, 2013; Turnbull et al., 2016). EPS protect bacterial cells from damage or death caused by surfactants, biocides, grazing predators and host defenses (Wagner and Iglewski, 2008; Flemming and Wingender, 2010).

Extracellular DNA plays an adhesive role in the initial stages and development of *P. aeruginosa* biofilms (Wagner and Iglewski, 2008; Mulcahy et al., 2010; Turnbull et al., 2016). The source of eDNA has been proposed to be due to either prophage-induced cell lysis or the release of membrane vesicles which contain DNA (Kadurugamuwa and Beveridge, 1995; Turnbull et al., 2016). Investigations into *P. aeruginosa* eDNA release by Turnbull et al. (2016) suggests explosive cell lysis-mediated MV production in biofilms and planktonic cultures are independent of the Pseudomonas Quinolone Signal.

Extracellular DNA in CF lungs is derived from both microorganisms, and also lysed host cells (Finkel and Kolter, 2001; Haussler and Fuqua, 2013). The concentration of eDNA in the CF lung can be as high as 14 mg/ml (Finkel and Kolter, 2001; Das et al., 2015) and could serve as an abundant nutrient source for bacterial growth (Finkel and Kolter, 2001; Haussler and Fuqua, 2013). *P. aeruginosa, Escherichia coli*, and *Shewanella* spp. have all been shown to be able to utilize DNA as a nutrient source (Finkel and Kolter, 2001; Pinchuk et al., 2008; Mulcahy et al., 2010; Lewenza, 2013).

**FIGURE 1:**
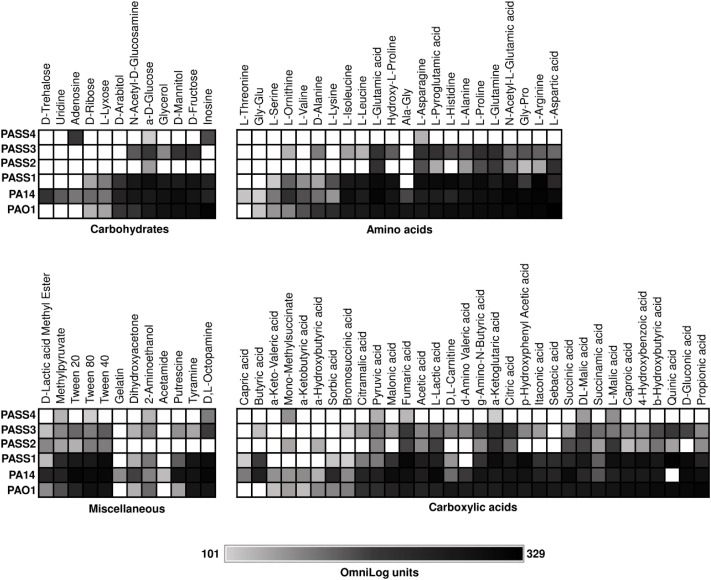
The catabolic phenome of *P. aeruginosa* wild-type strains. The maximal kinetic curve height of *P. aeruginosa* wild-type strains is expressed as a grayscale ranging from 101 (gray) to 329 (black) OmniLog units following growth at 37°C for 48 h. Phenotypes <101 OmniLog units (white) showed no detectable respiration.

We recently described *P. aeruginosa* CF isolates (PASS1-4) that showed significant variability in colonization and virulence-related traits (Penesyan et al., 2015). To investigate the metabolic capabilities of these strains, their ability to respire on 190 carbon sources was tested (Figure 1). One of the strains examined, PASS4, had lost the ability to utilize a broad range of carbon sources, and strong respiration was only observed on the purines inosine and adenosine. We found that PASS4 grew better on eDNA compared with other *P. aeruginosa* strains such as PAO1, suggesting that this strain may have become specialized for growth on DNA in the CF lung. Transcriptomic and proteomic analysis of PASS4, and genome sequencing of PASS4 mutants were undertaken to investigate the molecular basis of this metabolic specialization.

## Results and Discussion

### Phenotypic Comparison of *P. aeruginosa* CF Isolates PASS1-4

We used Biolog Phenotype Microarrays to compare the carbon utilization profiles of four *P. aeruginosa* CF isolates (PASS1-4) with two *P. aeruginosa* model strains (PAO1 and PA14). Strains were screened for their ability to utilize 190 sole carbon sources, including a range of carbohydrates, amino acids, carboxylic acids and miscellaneous compounds (Bochner et al., 2001). *P. aeruginosa* strains PAO1, PA14, and PASS1 had similar metabolic fingerprints. The other three CF isolates (PASS2-4) displayed reduced capabilities for carbon utilization (Figure 1). In particular, PASS4 showed a striking reduction in catabolic capability, only showing strong respiration on two compounds, the nucleosides adenosine and inosine. The capacity of PASS4 to grow on nucleosides was confirmed by culturing PASS4 on solid M9 minimal medium supplemented with 13 mM adenosine as a sole carbon source.

The carbon utilization profiles of PASS2-4 suggest that reduction in metabolic capabilities might be a common adaptation for *P. aeruginosa* in the CF lung. Biolog phenotype testing of 35 *P. aeruginosa* CF isolates in a previous study showed that metabolic reduction was common in CF isolates, with extensive heterogeneity amongst substrate utilization profiles (Jorgensen et al., 2015).

We hypothesized that PASS4 might be specialized to grow on DNA, which can be readily found in the CF lung environment. To investigate this possibility, PASS4 and PAO1 were grown in M9 minimal medium supplemented with 1.5 mg/ml of salmon sperm DNA (Sigma, United States) as a sole carbon source. Based on optical density measurement at OD_600_, the total biomass of PASS4 cells was 18% greater than PAO1 following growth in DNA over 7 days with constant aeration at 37°C. Dandekar et al. (2012) have observed the strain PAO1 to have a doubling time greater than 40 h when grown in adenosine as a sole carbon source (Dandekar et al., 2012). Both strains tested negative for production of extracellular DNase, suggesting that they uptake DNA where it is subsequently degraded intracellularly.

### The Transcriptome and Proteome of PAO1 and PASS4 Following Growth in DNA

The global transcriptional response and protein abundance of *P. aeruginosa* strains PASS4 and PAO1 grown to an OD_600_ = 0.6 with DNA as a sole carbon source was assessed using RNA sequencing (Table 1) and whole-cell proteomics. Growth on asparagine was used as a control, since this was one of the few carbon sources that PASS4 was able to utilize, according to the Biolog Phenotype Microarray analysis (Figure 1).

**Table 1:** Summary of *P. aeruginosa* PAO1 and PASS4 mapped reads following growth in DNA or asparagine.

			Percentage
Condition	Replicate	Paired reads	aligned
PAO1 grown in Asparagine	1	14532003	98.80
	2	11886289	98.77
	3	12821286	98.95
PAO1 grown in DNA	1	13805330	80.69
	2	12869272	89.53
	3	12660666	89.75
PASS4 grown in Asparagine	1	11436333	97.95
	2	13152398	97.08
	3	11153482	97.96
PASS4 grown in DNA	1	11846213	92.70
	2	11599891	91.39
	3	12826627	86.20


**FIGURE 2:**
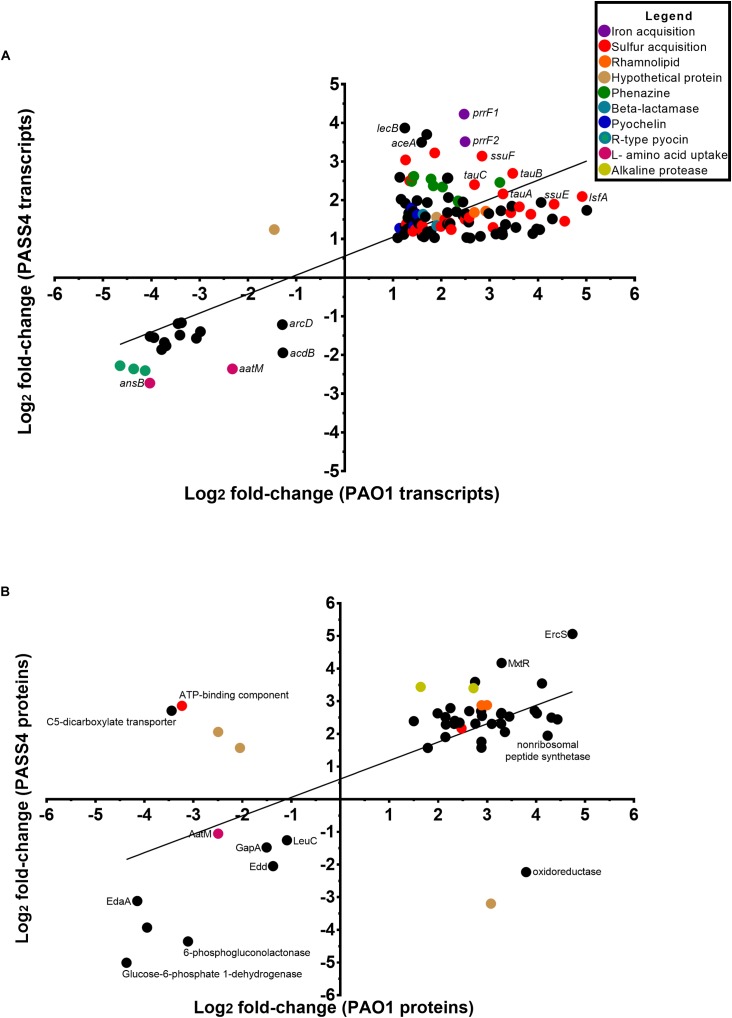
Comparison of the changes in expression at a transcript and protein level in *P. aeruginosa* PASS4 and PAO1 following growth in DNA; growth in asparagine was used as a reference. **(A)** The chart shows log_2_ fold changes of transcript data from RNA-sequencing (*P* < 0.01, log_2_ fold-change 1< to <–1). Correlation of PASS4 and PAO1 transcript data was moderately similar (*R*^2^ = 0.5983). **(B)** The chart shows log_2_ fold changes of shotgun protein data (*P* < 0.05, log_2_ fold-change 1< to <–1). Correlation of PASS4 and PAO1 protein data was moderately similar (*R*^2^ = 0.4072).

The transcriptomic analysis identified a total of 576 genes that were differentially transcribed by PASS4 when grown in the presence of DNA (*P-*value < 0.01, log_2_ fold-change 1< to <-1), with 322 genes upregulated and 254 genes downregulated (Supplementary Figure S1 and Supplementary Table ST1). There were a total of 423 genes differentially expressed by PAO1 when grown in DNA (*P-*value <0.01, log_2_ fold-change 1< to <-1), with 359 genes upregulated and 64 genes downregulated (Supplementary Figure S2 and Supplementary Table ST1). The transcriptional response of PASS4 and PAO1 grown in DNA in comparison to growth in asparagine had a correlation value of *R*^2^ = 0.5983 (Figure 2A). A total of 129 transcripts displayed similar expression patterns in both organisms, with 112 being upregulated and 17 downregulated (Supplementary Table ST1). Among the most noticeable differences, the two strains displayed differential expression of a hypothetical protein (PA3783), which was downregulated 3-fold in PAO1 and upregulated 4-fold in PASS4 during growth in DNA.

The shotgun proteomic analysis detected a total of 1962 proteins in PASS4, of which 307 displayed significant differential abundance between the two conditions (*P* < 0.05, log_2_ fold-change 1< to <-1) (Supplementary Figure S3 and Supplementary Table ST1). A total of 239 proteins showed greater abundance when the cells were grown on DNA as a sole carbon source in comparison to 68 proteins which displayed a decrease in abundance. A total of 2112 proteins were detected from PAO1, of 293 proteins were significantly differentially abundant (*P* < 0.05, log_2_ fold-change 1< to <-1), with an increase in abundance of 232 and decrease in abundance of 61 proteins (Supplementary Figure S4 and Supplementary Table ST1). A correlation analysis of the protein expression of PAO1 and PASS4 grown in DNA presented an *R*^2^ value of 0.4072 (Figure 2B). Only six proteins showed significant differences in protein abundance between PAO1 and PASS4 (Figure 2B), among those a C5 dicarboxylate transporter showed an 6.5-fold increase in abundance in PAO1 but showed a 10.9-fold decrease in abundance in PASS4 when these strains were grown in DNA.

### Increased Expression of Iron and Sulfate Acquisition Genes in Response to DNA

**FIGURE 3:**
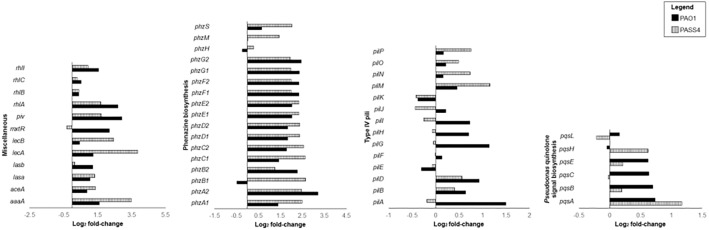
Differential gene expression (log_2_ fold-change) of virulence genes when *P. aeruginosa* strains PASS4 and PAO1 were grown in DNA compared to asparagine.

The pyoverdine (*pvd*) and pyochelin (*pch*) biosynthetic gene clusters encode the two major *P. aeruginosa* siderophores (Yeung et al., 2014; Lee and Zhang, 2015). Expression of the *pch* gene cluster and pyochelin receptor (*fptA*) was significantly induced by exposure to DNA in both PAO1 and PASS4 (Figure 3). The *pvd* gene cluster was significantly upregulated by DNA in PAO1, but not in PASS4 (Figure 3). PASS4 grown in DNA displayed a significant increase in the abundance of the PvdE and PvdF proteins. The iron-responsive small RNAs PrrF1 and PrrF2 (Wang et al., 2012) were upregulated in both PASS4 (11-fold and 19-fold, respectively) and PAO1 (both by 6-fold) (Figure 3). During iron limitation, these small RNAs enable inhibition of genes that encode “non-essential” iron-containing proteins (Smith, 2015). Additionally, PASS4 grown in DNA displayed an increased abundance of iron acquisition proteins including the putative TonB-dependent receptor family protein (PA0781), TonB-dependent siderophore receptor (PA4837), FeoB Fe^2+^ transporter (PA4358), FecA ferric citrate receptor (PA3901) and FiuR iron transport protein.

DNA as a sole carbon source led to the upregulation of an array of genes involved in sulfate metabolism in both PASS4 and PAO1. For example, the *tauABC* genes, encoding an ABC-transporter for the sulfate-containing amino acid taurine were upregulated more than 2- fold in both PASS4 and PAO1. The *tauD* gene, encoding taurine dioxygenase was upregulated more than 2-fold and had an 8-fold increase in protein abundance in PASS4. The expression of (*tauABCD*) is known to be regulated by sulfate starvation (van der Ploeg et al., 1996). Expression of other genes involved in the utilization of alternate sulfate sources, such as alkane sulphonates, were also increased in the presence of DNA. The *lsfA* gene encoding a thio-specific antioxidant was upregulated 4-fold in PASS4 and 30-fold in PAO1 following growth in DNA.

Sulfate regulatory systems also showed significant changes in gene expression in DNA grown cells. The ECF sigma factor PA2093 was 8-fold upregulated by DNA in PASS4. This sigma factor has previously been shown to be upregulated by sulfate limitation (Tralau et al., 2007). Typically, the TonB-dependent transducers PA2089 and PA2590 (both have increased protein abundance and significant upregulation of transcripts in PASS4 grown in DNA) sense an extracellular signal which is transmitted via the anti-sigma factor leading to the activation of ECF sigma factor (PA2093) and to the subsequent transcription of target genes such as PA2090 (upregulated significantly in PAO1 and PASS4 grown in DNA). The putative sulphonatase (PA2090) is part of a predicted extracellular sulfate (ECS) locus (PA2083–PA2094) (Robinson, 2013). All members of the ECS locus were upregulated at the transcriptional level in PASS4, with some upregulated in PAO1. The protein abundance of members of the ECS locus was also increased in PASS4.

During growth, most heterotrophic bacteria are known to maintain specific elemental ratios of carbon, nitrogen, sulfate, phosphorus and iron (Gray, 2017). Compared to most other carbon sources, DNA is a rich source for nitrogen and phosphorus. Thus, the increased expression of genes for iron and sulfate acquisition may reflect an attempt by *Pseudomonas* cells to balance their elemental ratios in a phosphorus/nitrogen rich setting.

### Increased Expression of Virulence Related Genes in Response to eDNA

The set of genes that were induced at least 4-fold by DNA in both PAO1 and PASS4 included two clusters of phenazine biosynthesis genes *phzA1B1C1D1E1F1G1* and *phzA2B2C2D2E2F2G2* (Figure 3). Several genes from the *phz1* and *phz2* gene clusters had significant increases in protein abundance in PAO1. Phenazine redox pigments such as pyocyanin and 1-hydroxyphenazine are important virulence factors: 1-hydroxyphenazine constrains mammalian cell respiration; and pyocyanin hinders epidermal cell growth and plays a role in acquisition of iron from transferrin (Wilson et al., 1988). Pyocyanin has also been shown to bind directly to the phosphate backbone of DNA, altering the viscosity of DNA solutions. During biofilm growth, by interacting with DNA, pyocyanin can facilitate electron transfer through DNA for maintenance of redox homeostasis between anoxic and oxygenated regions of the biofilms (Kempes et al., 2014; Das et al., 2015).

The RhlR quorum-sensing system regulates the phenazine biosynthesis and receptor genes (Whiteley et al., 1999; Reis et al., 2011), elastase *lasB*, protease *lasA*, and rhamnolipid biosynthesis genes *rhlAB* (Pearson et al., 1997; Grosso-Becerra et al., 2014). The *rhlR* transcripts were upregulated in both PAO1 and PASS4 grown in DNA, *rhlAB* transcripts and proteins showed increased abundance in both PASS4 and PAO1 when grown on DNA (Figure 3). The *lasA* and *lasB* transcripts were upregulated in PASS4 and PAO1 grown in DNA, the protein abundance of LasA was significantly increased in PASS4 and LasB was significantly increased in PAO1.

The *mxtR* (PA3271) gene encoding a sensor kinase was upregulated 5-fold in PASS4. The MxtR protein showed 8-fold and 16-fold increase in abundance in PAO1 and PASS4, respectively. MxtR has been shown to modulate the production of interbacterial 2-alkyl-4(1*H*)-quinolone (AQ) signal molecule via the LysR-type transcriptional regulator MexT (Zaoui et al., 2012) (no significant differential expression observed in our study). MxtR-induced AQ has an influence on the *P. aeruginosa* regulatory network, including the transcription of virulence genes coding for pyocyanin and rhamnolipids (Zaoui et al., 2012). PAO1 grown in DNA showed increased abundance of the PqsA, PqsE, and PqsL proteins (13-fold, 15-fold, and 3-fold, respectively). The *pqsABCDE* operon is positively regulated by the expression of 2-heptyl-4-hydroxyquinoline (HHQ) and is required for AQ biosynthesis (Rampioni et al., 2016). Although *pqsE* does not play a role in AQ biosynthesis, it has been shown to influence the production of virulence factors such as pyocyanin, phenazines and rhamnolipids (Heeb et al., 2011).

A QS dependent lysine-specific endoprotease (Oh et al., 2017), *piv* was upregulated 4.5-fold when PAO1 grown in DNA. This protease has been shown to cause killing of *Tenebrio molitor* larvae within 4 days post infection (Park et al., 2014). PIV also play a role in scavenging for nutrients (Barbier et al., 2014).

The *lecB* gene, encoding the fucose-binding lectin, showed 15-fold higher expression in PASS4 grown on DNA, but only two-fold increased expression in PAO1 (Figure 3). The LecB lectin has been shown to have cytotoxic effects on host respiratory epithelial cells, and plays an important role in facilitating adhesion to the airway mucosa. The *lecA* gene encoding a galactose-binding lectin was not significantly differentially expressed in either PAO1 or PASS4 when grown in DNA. This was surprising, as a previous study (Kohler et al., 2005) has shown the induction of the *lecA* lectin by adenosine.

The type 4 pilus (T4P) transcripts *pilA*, *pilG*, and *pilZ* were upregulated in PAO1 grown in DNA. Following growth of PASS4 in DNA there was has an increase in expression of the *pilM* transcript and an increase in protein abundance of PilH and PilM. The T4P plays a role in cell adhesion, host cell invasion (Hahn, 1997), biofilm formation, as well as DNA uptake (Mattick, 2002; Craig and Li, 2008).

The *aceA* gene encoding isocitrate lyase (Hogardt and Heesemann, 2010), which is specific to the glyoxylate shunt pathway, is 11-fold upregulated in PASS4 and 3-fold upregulated in PAO1 when grown in DNA. The glycoxylate shunt is known to be upregulated under conditions of oxidative stress, antibiotic stress, and host infection (Ahn et al., 2016). The *aceA* gene has been shown to be critical for *P. aeruginosa* infection in an alfalfa seedling model (Lindsey et al., 2008).

An arginine-specific autotransporter, AaaA showed an 11-fold higher protein abundance, and 2-fold downregulation of the transcript in PAO1. Previously, a gene knockout of *aaaA* led to attenuation of *P. aeruginosa* in a mouse chronic wound infection suggesting it plays a role in virulence (Luckett et al., 2012).

### Other Transcriptional Changes in Response to DNA

As expected, the *ansB* gene, encoding L-asparaginase, showed 16-fold and 7-fold decrease in gene expression in DNA-grown cells compared with asparagine-grown cells for PAO1 and PASS4, respectively. Other genes in the same regulon as *ansB*, such as the *aatM* gene, encoding for acidic L-amino acid uptake, were also upregulated by asparagine. The branched chain amino acid catabolism genes *liuA, liuB, liuC, bkdA1, bkdA2, bkdB, lpdV, mmsB*, and *mmsA* were upregulated in asparagine-grown cells, suggesting a regulatory link between asparagine and branched chain amino acid degradation. The transport and utilization of branch chain amino acids, arginine and ornithine has been reported to be governed by the CbrAB/Crc regulatory system (Sonnleitner et al., 2012).

The PA0622–PA0624 genes involved in the production of the R-type pyocin were downregulated by a more than 4-fold in both PAO1 and PASS4 during growth in DNA compared to asparagine. These genes, along with other genes from the R- and F-pyocin gene cluster, encode a prophage endolysin which is essential for explosive cell lysis that leads to increased availability of public goods such as cytosolic proteins, eDNA, and membrane vesicles (Turnbull et al., 2016). The transcriptomic data suggests that the concentration of eDNA is an important factor regulating the expression of genes controlling explosive cell lysis.

### Carbon Utilization by *P. aeruginosa* Mutants

The transcriptomic and proteomic analyses of PASS4 grown on DNA identified a broad range of genes whose expression might be regulated by DNA including many virulence factors. However, it did not provide any clear answers as to the molecular basis of metabolic specialization in PASS4. To further investigate the specialization of PASS4 for growth on DNA, we screened mutants of PASS4 to isolate strains that had broader substrate utilization capabilities. To obtain such mutants, we inoculated *P. aeruginosa* PASS4 on minimal M9 medium containing 20 mM *N*-Acetyl-D-glucosamine (GlcNAc), a carbon source that PASS4 cells were unable to utilize (Figure 1). Within 7 days, spontaneous mutant colonies were observed and subsequently cultured into liquid M9 minimal salts medium containing 20 mM GlcNAc. This culture was consecutively subcultured for 14 days until cells reached an OD_600_ = 0.7 within 35 h. At the end of this process, we obtained eight *P. aeruginosa* PASS4 mutants which had gained the ability to grow on GlcNAc.

**FIGURE 4:**
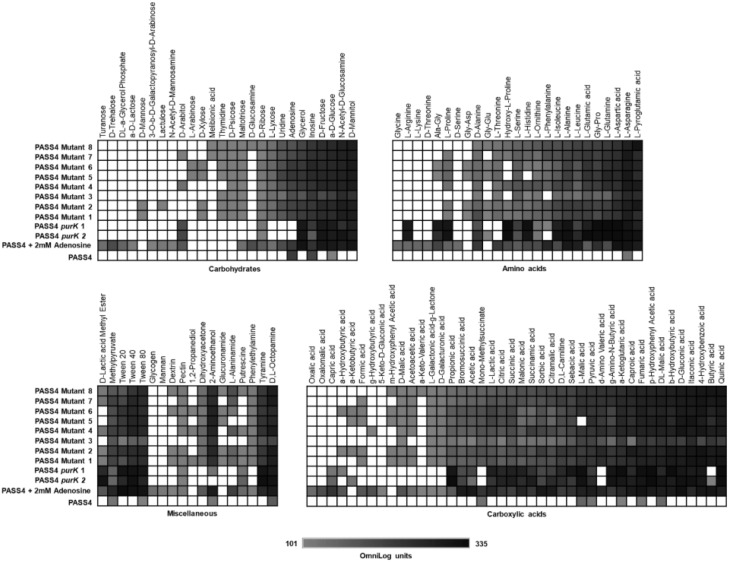
The catabolic phenome profile of *P. aeruginosa* PASS4 mutants, PASS4 supplemented with 2 mM adenosine and PASS4 complemented with *purK* compared to *P. aeruginosa* PASS4 wild type as control. The maximal kinetic curve height difference to *P. aeruginosa* wild-type strain PASS4 is expressed as a grayscale ranging from 101 (gray) to 335 OmniLog units (black). White represents no detectable respiration.

The eight mutants displayed much broader carbon catabolic profiles than the parental PASS4 strain when tested on Biolog Phenotype Microarray plates. All of these mutants had gained the ability to utilize a wide range of carbon sources including amino acids, carbohydrates and carboxylic acids (Figure 4). The substrate utilization profiles of the mutants resembled those of most *P. aeruginosa* strains, including PAO1, PA14, and PASS1. This dramatic change in carbon utilization capability suggests that the mutation(s) in these PASS4 mutants had apparently circumvented the genetic specialization in the parental PASS4 isolate that allowed it to grow well on DNA, but not on most other carbon sources.

### PASS4 Mutants Have Mutations in the *purK* Gene

Genome sequencing was undertaken on the Miseq v2 platform of eight PASS4 mutants that had gained the ability to utilize GlcNAc and an array of other carbon sources. In parallel, we also resequenced the PASS4 genome as a reference. Bioinformatic analysis identified single nucleotide polymorphisms (SNPs) between the mutants and the PASS4 genome. All of the PASS4 mutants contained a mutation in the *purK* gene, located at codon 214 (for PASS4 mutants 2 and 3) or codon 354 (for PASS4 mutants 1 and 4 to 8) (Supplementary Table ST2 and Supplementary Figure S5). Additional SNPs were also detected in mutants 1 and 7, in a chemotaxis transducer (*pctB*) and a hypothetical protein, respectively. The *purK* gene encodes a subunit of the phosphoribosylaminoimadazole carboxylase enzyme, a critical step in the *de novo* synthesis of purines (Zhang et al., 2008). This suggests that PASS4 is defective in purine biosynthesis, which explains why it grows well only on DNA and purines, while growing poorly, or not at all, on essentially all other tested carbon sources, as would be expected for a purine auxotroph.

To test whether the inability of PASS4 to grow on various carbon sources was due to a defect in purine biosynthesis, we repeated the Biolog phenotype testing but including supplementation with 2 mM adenosine. This concentration of adenosine was not sufficient as a sole carbon source to support the growth of PASS4. The supplementation of 2 mM adenosine and complementation of PASS4 with *purK* enabled PASS4 to grow on a wide range of carbohydrates, amino acids, and carboxylic acids that it previously was unable to utilize (Figure 4). This provides evidence that the underlying growth defect in PASS4 is due to a defect in *de novo* purine biosynthesis. Since all of the PASS4 mutants had changes within the *purK* gene, it is highly likely that PurK is defective in PASS4.

The CF lung environment contains high concentrations of eDNA. The defect in purine biosynthesis in PASS4, coupled with an ability to grow faster on DNA as a sole carbon source compared with other *P. aeruginosa* strains, probably reflects a niche adaptation to this DNA-rich environment. Species from the genera *Chlamydia* and *Rickettsia*, the parasitic flagellate protozoa *Trypanosoma*, *Treponema pallidum*, *Mycoplasma*, *Ureaplasma*, *Mesoplama*, *Borrelia*, and even *Lactobacillus*, all appear to exist without the presence of the classical *de novo* purine nucleotide biosynthesis pathway (Zhang et al., 2008). These organisms are either obligate parasites or are associated with mucosal epithelial layers of the host (Zhang et al., 2008). Scavenging purines or nucleic acids from their host may be a common adaptation in many parasites, pathogens and commensals.

The PASS4 strain showed modest growth on asparagine as a sole carbon source. Indeed, this compound was used as a growth nutrient in the transcriptomic and proteomic experiments in this study. Asparagine is required for the conversion of inosine monophosphate to adenylosuccinate, in the first enzymatic step in dATP synthesis after *de novo* purine biosynthesis (Supplementary Figure S6). The ability of PASS4 to grow on asparagine as a sole carbon source could be due to high levels of asparagine enabling sufficient flux through the purine biosynthesis pathway to allow growth. In turn, this suggests a decrease in the efficiency of the PurK enzyme in PASS4, rather than a complete loss of function.

## Conclusion

Phenotypic analysis revealed that the *P. aeruginosa* CF isolate PASS4 was only able to grow on a limited range of carbon sources, including purines and DNA. On these substrates it showed higher growth rates than other *P. aeruginosa* isolates. This suggested that this strain is specialized to live on eDNA available in the CF lung. We investigated the genetic basis of this apparent metabolic specialization. Transcriptomic and proteomic studies of *P. aeruginosa* PASS4 and PAO1 grown in DNA revealed that eDNA affected the expression of many genes, particularly virulence and host-adaptation genes. This suggests that eDNA may be an important signal in the CF lung for expression of virulence factors by *P. aeruginosa*.

The transcriptomic and proteomic expression analyses did not provide any direct insights into the metabolic specialization of PASS4. Genome sequencing of PASS4 mutants that had gained the ability to catabolize GlcNAc, as well as a wide range of unrelated carbon sources, indicated that they all contained mutations in the *purK* gene, which encodes a key enzyme in the *de novo* purine biosynthesis pathway. This suggested that PASS4 was a purine auxotroph, and this was confirmed by phenotypic testing showing addition of 2 mM adenosine could rescue growth of PASS4 on a broad range of carbon sources. Purine auxotrophy may represent a viable microbial strategy for adaptation to DNA-rich environments such as the CF lung.

## Materials and Methods

### *Pseudomonas aeruginosa* Strains and Media

*Pseudomonas aeruginosa* strains used for this study were PAO1 (Stover et al., 2000), PA14 (He et al., 2004) and PASS1-4 (Penesyan et al., 2015; Thaysen-Andersen et al., 2015). The PASS1-4 isolates were obtained from the sputum of CF patients at the Westmead Hospital (Sydney, Australia). The *P*. *aeruginosa* isolate PASS1 was obtained from a 40-year old female patient, PASS2 from a 27-year old male, PASS3 from a 23-year-old male and PASS4 from a 23-year old female (Penesyan et al., 2015; Thaysen-Andersen et al., 2015). These isolates were maintained in glycerol stocks at -80°C. *P. aeruginosa* isolates were routinely cultured on Luria Bertani (LB) media, solid or liquid, from the frozen stock, allowing minimum passages during cultivation.

### Production of Exoenzyme DNase

The test for the production of exoenzyme DNase by *P. aeruginosa* PAO1 and PASS4 was conducted with a use of DNase test agar (Oxoid). Briefly, as per manufacturer’s instructions, the strains were streaked on a DNase test agar plate and incubated at 37°C for 24 h. The DNase test agar contained tryptose, DNA and NaCl. The production of DNase leads to the hydrolysis of DNA in the media. Therefore, following incubation, the plates were flooded with 1N HCl to observe for a clearing/hydrolysis of DNA by the bacterium.

### RNA Extraction and RNA-Seq Transcriptomics

*Pseudomonas aeruginosa* strains PAO1 and PASS4 were simultaneously inoculated into 5 ml of LB liquid medium from the frozen stock and grown overnight. Overnight cultures were used to inoculate 15 ml of M9 minimal medium supplemented with either L-Asparagine (20 mM) or DNA (1.5 mg/ml) (Salmon sperm DNA, Sigma, United States), both in biological triplicates. Cultures were grown until mid-exponential phase (OD_600_ = 0.6). RNA was extracted from these cultures using the miRNEasy RNA extraction kit (Qiagen) according to the manufacturer’s protocol. To remove any residual genomic DNA, the samples were treated with DNAse using the TURBO DNAse kit (Invitrogen, United States). The quality and quantity of extracted RNA was assessed on a NanoDrop spectrophotometer. To remove highly abundant ribosomal RNA from the RNA extracts before sequencing, the samples were treated using RiboZero GN Magnetic rRNA depletion kit (Epicenter). The rRNA depleted samples were purified using RNeasy MinELute Cleanup kit (Qiagen, Germany) and re-assessed on NanoDrop and submitted to the Ramaciotti Centre for Genomics for paired-end RNA Sequencing on the HiSeq 2000 platform. Paired-end RNA Sequence files obtained from Ramaciotti were assessed for quality using FastQC software (Babraham Bioinformatics, United Kingdom) and processed by trimming the first 10 nucleotides using Fastx Toolkit. Trimmed sequences were tiled against the complete genome of PAO1 via EdgePro software (Magoc et al., 2013) and differential expression calculated using DESeq software (Anders and Huber, 2010). *De novo* transcript assembly and differential gene expression analysis was performed for PASS4 strain using the Rockhopper 2.03 tool (McClure et al., 2013; Tjaden, 2015) to supplement the data on PASS4 genes absent in PAO1 genome. The RNA-seq raw data reported here are accessible under the Gene Expression Omnibus submission accession number GSE100287.

### Protein Extraction and Label-Free Shotgun Proteomics

*Pseudomonas aeruginosa* strains PAO1 and PASS4 were inoculated into M9 Minimal salts media with 100 μM calcium chloride (CaCl_2_) and 2 mM magnesium sulfate (MgSO_4_) containing either L-Asparagine (20 mM) as a control condition or DNA (1.5 mg/ml) as an experimental condition. Cultures were grown aerobically (*n* = 3) at 37°C with constant shaking at 200 rpm. Overnight cultures were subcultured into the same respective medium, prior to harvesting cells in logarithmic phase (OD_600_ = 0.6). For proteomic analysis, cells were harvested from 60 ml of culture by centrifugation at 3220 *g*, 10 min at 4°C (Beckman centrifuge, United States). Cells were washed with phosphate buffered saline (pH 7.4) and stored at -80°C until further processing. Proteins were extracted by lysing cell pellets with sodium dodecyl sulfate (SDS) lysis buffer (2.3% w/v SDS, 0.12 M Tris, 0.4 mM EDTA, 4% w/v glycerol and 0.05% v/v β-mercaptoethanol (pH 6.8). Followed by four 30 s rounds of bead beating at 5.5 m/sec (FastPrep FP120, United States), with intermittent cooling. Cellular debris was removed by centrifugation at for 10,000 × *g* for 10 min (Eppendorf, model 5804R) and the supernatants were collected and stored at -20°C for further processing. The extracted proteins were precipitated using a methanol/chloroform/water protocol (Wessel and Flugge, 1984). Resultant proteins pellets were resuspended in 1% w/v SDS, 13% w/v glycerol and 33 mM Tris (pH 6.8) and quantitated using a BCA Protein assay kit as per manufacturer’s instructions (Thermo Fisher Scientific, United States). 30 μg of each sample was diluted with sample loading buffer, [1% w/v SDS, 13% w/v glycerol and 33 mM Tris, 20 mM β-mercaptoethanol, 0.004% bromophenol blue (pH 6.8)], denatured by boiling (95°C, 4 min) and separated using SDS-PAGE (Bio-Rad, Australia). After electrophoresis, proteins were visualized using colloidal Coomassie Blue and processed further for tryptic digestion as detailed in Mirzaei et al. (2012). Briefly, each gel lane corresponding to individual sample was cut into 16 pieces, chopped and placed into a well of a 96-well plate. The gel pieces were briefly washed with 100 mM NH_4_HCO_3_, followed by washing twice with acetonitrile (ACN) (50%)/100 mM NH_4_HCO_3_ (50%) for 10 min. Finally, gel pieces were dehydrated with 100% ACN and air-dried. Proteins were reduced with 10 mM dithiothreitol (DTT) in NH_4_HCO_3_ (50 mM) at 37°C for 1 h, followed by alkylation with 50 mM iodoacetamide in NH_4_HCO_3_ (50 mM), in the dark at RT for 1 h. Samples were then washed with 100 mM NH_4_HCO_3_, followed by ACN (50%)/100 mM NH_4_HCO_3_ (50%) for 10 min, dehydrated with 100% ACN and then air-dried. Finally, samples were digested with 20 μL of trypsin (12.5 ng/mL 50 mM NH_4_HCO_3_), overnight at 37°C. Proteolytic peptides were extracted twice with ACN (50%)/formic acid (2%), dried using a vacuum centrifuge and reconstituted to 10 μL with 2% formic acid for LC-MS/MS analysis.

Peptides were analyzed using a Q Exactive Hybrid Quadrupole-Orbitrap mass spectrometer coupled to a high pressure liquid chromatography unit (Thermo Fisher Scientific, United States). Peptides were separated on a 60-min reverse phase gradient of 1–50% solvent B (acetonitrile in 0.1% formic acid) gradient. In each data collection cycle, one full MS scan (350–2000 m/z) was recorded in the Orbitrap. Subsequently, MS2 analysis was conducted for top 10 most intense ions and were fragmented by higher-energy collisional dissociation (HCD) with following settings; normalized collision energy of 30%, isolation window 3.0 m/z, maximum ion accumulation time 60 ms with a dynamic exclusion for 10 s.

Protein identification and quantification was performed on Proteome Discoverer 1.3 (Thermo Fisher Scientific) using Mascot search engine (Perkins et al., 1999). *P. aeruginosa* strain PAO1 protein sequence database retrieved from GenBank (January 2013) and *Pseudomonas* Genome Database^1^ and *in-silico* translated genome databases of PASS4 (Penesyan et al., 2015) were used as the search databases. Database searching against the decoy database was also performed to evaluate the false discovery rate (FDR) of peptide identification. All searches were performed using a static modification for cysteine alkylation and methionine oxidation, acetylation (protein N-term) as dynamic modifications, precursor ion tolerance of 10 ppm and a fragment ion tolerance of 0.02 Da were used. Peptide matches were filtered with peptide and protein FDR < 1%. Then for each identified peptide, its abundance (peak area) was estimated by calculating the area under the extracted ion chromatograms (XIC) curve.

The mass spectrometry proteomics data has been deposited to the ProteomeXchange Consortium via the PRIDE partner repository (Vizcaíno et al., 2014) with the dataset identifier PXD006742.

For statistical analyses, log-transformed quantitative values were used to conduct two-sample *t*-tests comparing protein expression of PASS4 or PAO1 in DNA relative to L-Asparagine using the in-house developed program based on R modules (Mirzaei et al., 2012; Neilson et al., 2014). Proteins with log fold change ± 1 and *t*-test *p*-value < 0.05 were considered to be significantly differentially expressed.

### Functional Analysis

The transcripts and proteins were mapped to virulence factor annotations and to Cluster of Orthologous Groups (COG) categories obtained from the Pseudomonas Database (Winsor et al., 2016).

### Generation of *P. aeruginosa* PASS4 Mutants

Single colonies of *P. aeruginosa* strain PASS4 were inoculated into 10 ml of LB liquid medium, in triplicate, and grown overnight with constant shaking of 200 rpm at 37°C. Overnight cultures were centrifuged at 4000 × *g* for 7 min at 4°C in an Eppendorf centrifuge Model 5430. Once the supernatant was discarded the cells were washed with phosphate buffered saline and spun at 4000 *g* for 7 min at 4°C. The cells were then resuspended in 5 ml phosphate buffered saline and 2.04 × 10^8^ washed cells were spread on 20 mM GlcNAc. Once spontaneous mutant colonies were obtained, colonies were cultured into M9 minimal salts medium with 100 μM calcium chloride (CaCl_2_) and 2 mM magnesium sulfate (MgSO_4_) containing 20 mM GlcNAc. This culture was consecutively subcultured till cells were able to reach an OD_600_ = 0.7 within ∼35 h.

### Whole Genome Sequencing and SNP Analysis of PASS4 Mutants

Single colonies of 8 *P. aeruginosa* strain PASS4 mutants and the parental PASS4 strain were inoculated into 10 ml of LB liquid medium, in triplicates, from LB agar and grown overnight with constant shaking of 200 rpm at 37°C. DNA was extracted from the samples according to DNA Isolation DNeasy Blood and Tissue Kit protocol for Gram-negative bacteria (QIAGEN, Germany). Total DNA was quantified using the Nanodrop and submitted to the Ramaciotti Centre for Genomics for paired-end DNA Sequencing on MiSeq v2 platform. Paired-end DNA sequence files obtained from Ramaciotti were assessed for quality using FastQC software (Babraham Bioinformatics, United Kingdom). The pair-end reads for each sample were merged by FLASH-1.2.11 (Magoc and Salzberg, 2011). The merged reads were filtered with the FASTQ quality filter with minimum quality score being 20, and 90 being the minimum percent of bases with a quality score of 20. The reads were then trimmed with FASTQ quality trimmer ensuring minimum length of sequence of 200 and quality thresholds of nucleotide of 28. The FASTQ files were converted to FASTA and then subjected to SNP analysis according to the user guide of kSNP3 software (Gardner et al., 2015).

### Growth of *P. aeruginosa* PASS4 in Adenosine

To identify the lowest concentration of adenosine as a sole carbon source that supported growth of PASS4, growth assays were undertaken in liquid media in M9 minimal salt medium with 100 uM calcium chloride (CaCl_2_) and 2 mM magnesium sulfate (MgSO_4_) supplemented with 0.02 mM – 20 mM adenosine using a broth dilution method essentially as previously described (Wiegand et al., 2008). Cell growth was determined spectrophotometrically.

### Complementation of PASS4 Using the pME6032 Vector

*Pseudomonas aeruginosa* PASS4 was complemented with an intact *P. aeruginosa* PAO1 *purK* gene using pME6032 expression vector containing tetracycline resistance marker (Heeb et al., 2002). The pME6032 vector was kindly gifted by Professor Stephan Heeb, University of Nottingham, United Kingdom. Cloning was performed using the In-Fusion HD cloning kit (Takara Bio, United States) following the procedures suggested in the manufacturer’s protocol. Briefly, the *purK* gene was amplified via PCR using the Platinum SuperFi PCR Master Mix (Thermo Fisher Scientific) with *P. aeruginosa* PAO1 genomic DNA as a template, and the primer pair 2_purK_F/R (Table 2). The pME6032 plasmid was linearized using the EcoRI and XhoI restriction enzymes (New England Biolabs) and used in In-Fusion cloning reaction also containing the 5X In-Fusion HD Enzyme Mix and the purified *purK* PCR fragment. After completion, the reaction mix was used for transformation using *E. coli* Stellar Competent Cells (Takara Bio, United States) according to the manufacturer’s protocol. Petri dishes containing solid LB medium supplemented with 25 μg/ml of tetracycline were used to select and maintain the *E. coli* transformants. The vector with *purK* gene insertion was purified from the culture of *E. coli* cells using the Wizard Plus SV Minipreps DNA Purification System (Promega) and the manufacturer’s protocol and used to transform the wild type PASS4 strain via electroporation. Overnight culture of PASS4 was inoculated in 2 × 5 ml fresh LB broth in 50 ml Falcon tubes and incubated overnight, with shaking, at 42°C, following a previously described method (Kaur et al., 2015). After the incubation the cultures were combined, centrifuged (10 min at 5000 rpm) and the cell pellet resuspended in 5 ml fresh LB broth containing 6.5 U/ml of Alginate Lyase (Sigma) to break down the excess alginate. The mixture was incubated at 37°C for 40 min after which the cells were washed with 1 ml ice-cold MilliQ water and resuspended in 20 μl of sterile ice-cold water, and 1 μl of pME6032_*purK* plasmid was added (60 ng). The mixture was transferred to cold electroporation cuvette (2 mm gap, Bio-Rad) and electroporated using Bio-Rad MiniPulser (2.5 kV pulse). Immediately after applying the pulse, 1 ml fresh LB was added to the electroporation mixture, mixed, and incubated at 37°C for 3 h. After the incubation the mixture was serially diluted and plated on Petri dishes containing solid LB medium supplemented with 120 μg/ml of tetracycline. The presence of the *purK* gene in the PASS4 transformants was confirmed using a PCR with the insert_F/R primer pair (Table 2), as well as via Sanger sequencing using the same primers. The sequencing was performed at Macrogen, South Korea. Sequences obtained from Macrogen were aligned with the sequence of the *in silico* predicted product consisting of pME6032 insertion region and the full-length *purK* gene, yielding a 100% match.

**Table 2:** Primers used in PASS4 complementation experiments.

Primer name	Sequence (5′ to 3′)
2_*purK*_F	CAGGAAACAGAATTCATGAAAATCGGTGTCATCGGTGGC
2_*purK*_R	TAGTCCGAGGCCTCGAGTCACGCCTCGATCAGC
Insert_F	ATTCGTGTCGCTCAAGG
Insert_R	CTCGGGTAACATCAAGG


### Biolog Phenotype Microarray Analysis

Biolog Phenotype analysis was carried out for *P. aeruginosa* parental strain PASS4, 8 PASS4 mutants and PASS4 complemented with *purK*, using PM1 and PM2A MicroPlate^TM^ Carbon Source Phenotype Microarrays (Biolog, United States) containing a total of 190 substrates (including a range of carbohydrates, carboxylic acids, amino acids, fatty acids, amines, alcohols, polymers, amides and esters) and a negative control for each plate (Bochner et al., 2001). Bacterial cell suspensions (absorbance of 0.07 at 600 nm) were prepared in the inoculating fluid (IF-0a, Biolog, United States) and 100 ul of the inoculum was dispensed into each well of the plate using a multichannel pipette. After inoculation, the plates were incubated in the OmniLog incubator/reader (Biolog) for 48 h at 37°C. Cell respiration was recorded every 15 min by a charge-coupled device camera. The changes in the color of inoculated wells due to the conversion of the tetrazolium dye present in the wells into the purple derivative during cell respiration, were plotted over the whole period of incubation yielding kinetic curves representative of the metabolic activity of the strain in the presence of a particular carbon source. Raw values were imported from the OmniLog reader for heatmap generation. For the adenosine-supplemented experiments, *P. aeruginosa* PASS4 was suspended in inoculating fluid supplemented with 2 mM adenosine before dispensing 100 μl to each well of the PM1 and PM2A plates (Biolog, United States), which were subjected to Biolog Phenotype Microarray analysis as described above.

## Data Availability

The datasets generated for this study can be found in GENE EXPRESSION OMNIBUS, GSE100287.

## Author Contributions

All authors listed have made a substantial, direct and intellectual contribution to the work, and approved it for publication.

## Conflict of Interest Statement

The authors declare that the research was conducted in the absence of any commercial or financial relationships that could be construed as a potential conflict of interest.

## Footnotes

1 www.pseudomonas.com

## References

[B1] Ahn S. Jung J. Jang I. A. Madsen E. L. Park W. (2016). Role of Glyoxylate Shunt in Oxidative Stress Response. *J. Biol. Chem.* 291 11928–11938. 10.1074/jbc.M115.708149 27036942 PMC4882458

[B2] Anders S. Huber W. (2010). Differential expression analysis for sequence count data. *Genome Biol.* 11:R106. 10.1186/gb-2010-11-10-r106 20979621 PMC3218662

[B3] Barbier M. Damron F. H. Bielecki P. Suárez-Diez M. Puchałka J. Albertí S. (2014). From the environment to the host: re-wiring of the transcriptome of *Pseudomonas aeruginosa* from 22°C to 37°C. *PLoS One* 9:e89941. 10.1371/journal.pone.0089941 24587139 PMC3933690

[B4] Bochner B. R. Gadzinski P. Panomitros E. (2001). Phenotype microarrays for high-throughput phenotypic testing and assay of gene function. *Genome Res.* 11 1246–1255. 11435407 10.1101/gr.186501 PMC311101

[B5] Craig L. Li J. (2008). Type IV pili: paradoxes in form and function. *Curr. Opin. Struct. Biol.* 18 267–277. 10.1016/j.sbi.2007.12.009 18249533 PMC2442734

[B6] Dandekar A. A. Chugani S. Greenberg E. P. (2012). Bacterial quorum sensing and metabolic incentives to cooperate. *Science* 338 264–266. 10.1126/science.1227289 23066081 PMC3587168

[B7] Das T. Kutty S. K. Tavallaie R. Ibugo A. I. Panchompoo J. Sehar S. (2015). Phenazine virulence factor binding to extracellular DNA is important for *Pseudomonas aeruginosa* biofilm formation. *Sci. Rep.* 5:8398. 10.1038/srep08398 25669133 PMC4323658

[B8] Finkel S. E. Kolter R. (2001). DNA as a nutrient: novel role for bacterial competence gene homologs. *J. Bacteriol.* 183 6288–6293. 11591672 10.1128/JB.183.21.6288-6293.2001 PMC100116

[B9] Flemming H.-C. Wingender J. (2010). The biofilm matrix. *Nat. Rev. Microbiol.* 8 623–633. 10.1038/nrmicro2415 20676145

[B10] Folkesson A. Jelsbak L. Yang L. Johansen H. K. Ciofu O. Hoiby N. (2012). Adaptation of *Pseudomonas aeruginosa* to the cystic fibrosis airway: an evolutionary perspective. *Nat. Rev. Microbiol.* 10 841–851. 10.1038/nrmicro2907 23147702

[B11] Gardner S. N. Slezak T. Hall B. G. (2015). kSNP3.0: SNP detection and phylogenetic analysis of genomes without genome alignment or reference genome. *Bioinformatics* 31 2877–2878. 10.1093/bioinformatics/btv271 25913206

[B12] Gellatly S. L. Hancock R. E. W. (2013). *Pseudomonas aeruginosa*: new insights into pathogenesis and host defenses. *Pathog. Dis.* 67 159–173. 10.1111/2049-632X.12033 23620179

[B13] Gray N. (2017). *Water Science and Technology an Introduction.* 4th Edn Boca Raton, FL: CRC Press.

[B14] Grosso-Becerra M. V. Croda-García G. Merino E. Servín-González L. Mojica-Espinosa R. Soberón-Chávez G. (2014). Regulation of *Pseudomonas aeruginosa* virulence factors by two novel RNA thermometers. *Proc. Natl. Acad. Sci. U.S.A.* 111 15562–15567. 10.1073/pnas.1402536111 25313031 PMC4217398

[B15] Hahn H. P. (1997). The type-4 pilus is the major virulence-associated adhesin of *Pseudomonas aeruginosa*–a review. *Gene* 192 99–108. 9224879 10.1016/s0378-1119(97)00116-9

[B16] Haussler S. Fuqua C. (2013). Biofilms 2012: new discoveries and significant wrinkles in a dynamic field. *J. Bacteriol.* 195 2947–2958. 10.1128/JB.00239-13 23625847 PMC3697544

[B17] He J. Baldini R. L. Déziel E. Saucier M. Zhang Q. Liberati N. T. (2004). The broad host range pathogen *Pseudomonas aeruginosa* strain PA14 carries two pathogenicity islands harboring plant and animal virulence genes. *Proc. Natl. Acad. Sci. U.S.A.* 101 2530–2535. 14983043 10.1073/pnas.0304622101 PMC356984

[B18] Heeb S. Blumer C. Haas D. (2002). Regulatory RNA as mediator in GacA/RsmA-dependent global control of exoproduct formation in *Pseudomonas fluorescens* CHA0. *J. Bacteriol.* 4 1046–1056. 11807065 10.1128/jb.184.4.1046-1056.2002 PMC134805

[B19] Heeb S. Fletcher M. P. Chhabra S. R. Diggle S. P. Williams P. Cámara M. (2011). Quinolones: from antibiotics to autoinducers. *FEMS Microbiol. Rev.* 35 247–274. 10.1111/j.1574-6976.2010.00247.x 20738404 PMC3053476

[B20] Hogardt M. Heesemann J. (2010). Adaptation of *Pseudomonas aeruginosa* during persistence in the cystic fibrosis lung. *Int. J. Med. Microbiol.* 300 557–562. 10.1016/j.ijmm.2010.08.008 20943439

[B21] Jorgensen K. M. Wassermann T. Johansen H. K. Christiansen L. E. Molin S. Hoiby N. (2015). Diversity of metabolic profiles of cystic fibrosis *Pseudomonas aeruginosa* during the early stages of lung infection. *Microbiology* 161 1447–1462. 10.1099/mic.0.000093 25873584

[B22] Kadurugamuwa J. L. Beveridge T. J. (1995). Virulence factors are released from *Pseudomonas aeruginosa* in association with membrane vesicles during normal growth and exposure to gentamicin: a novel mechanism of enzyme secretion. *J. Bacteriol.* 177 3998–4008. 7608073 10.1128/jb.177.14.3998-4008.1995 PMC177130

[B23] Kaur J. Pethani B. P. Kumar S. Kim M. Sunna A. Kautto L. (2015). *Pseudomonas aeruginosa* inhibits the growth of Scedosporium aurantiacum, an opportunistic fungal pathogen isolated from the lungs of cystic fibrosis patients. *Front. Microbiol.* 6:866. 10.3389/fmicb.2015.00866 26379643 PMC4547459

[B24] Kempes C. P. Okegbe C. Mears-Clarke Z. Follows M. J. Dietrich L. E. (2014). Morphological optimization for access to dual oxidants in biofilms. *Proc. Natl. Acad. Sci. U.S.A.* 111 208–213. 10.1073/pnas.1315521110 24335705 PMC3890773

[B25] Kohler J. E. Zaborina O. Wu L. Wang Y. Bethel C. Chen Y. (2005). Components of intestinal epithelial hypoxia activate the virulence circuitry of *Pseudomonas*. *Am. J. Physiol. Gastrointest. Liver Physiol.* 288 G1048–G1054. 10.1152/ajpgi.00241.2004 15550562

[B26] Lee J. Zhang L. (2015). The hierarchy quorum sensing network in *Pseudomonas aeruginosa*. *Protein Cell* 6 26–41. 10.1007/s13238-014-0100-x 25249263 PMC4286720

[B27] Lewenza S. (2013). Extracellular DNA-induced antimicrobial peptide resistance mechanisms in *Pseudomonas aeruginosa*. *Front. Microbiol.* 4:21. 10.3389/fmicb.2013.00021 23419933 PMC3572637

[B28] Lindsey T. L. Hagins J. M. Sokol P. A. Silo-Suh L. A. (2008). Virulence determinants from a cystic fibrosis isolate of *Pseudomonas aeruginosa* include isocitrate lyase. *Microbiology* 154 1616–1627. 10.1099/mic.0.2007/014506-0 18524916

[B29] Luckett J. C. A. Darch O. Watters C. Abuoun M. Wright V. Paredes-Osses E. (2012). A novel virulence strategy for *Pseudomonas aeruginosa* mediated by an autotransporter with arginine-specific aminopeptidase activity. *PLoS pathog.* 8:e1002854. 10.1371/journal.ppat.1002854 22927813 PMC3426542

[B30] Magoc T. Salzberg S. L. (2011). FLASH: fast length adjustment of short reads to improve genome assemblies. *Bioinformatics* 27 2957–2963. 10.1093/bioinformatics/btr507 21903629 PMC3198573

[B31] Magoc T. Wood D. Salzberg S. L. (2013). EDGE-pro: estimated degree of gene expression in prokaryotic genomes. *Evol. Bioinform.* 9 127–136. 10.4137/EBO.S11250 23531787 PMC3603529

[B32] Mall M. A. (2008). Role of cilia, mucus, and airway surface liquid in mucociliary dysfunction: lessons from mouse models. *J. Aerosol. Med. Pulm. Drug Deliv.* 21 13–24. 10.1089/jamp.2007.0659 18518828

[B33] Mattick J. S. (2002). Type IV pili and twitching motility. *Annu. Rev. Microbiol.* 56 289–314. 12142488 10.1146/annurev.micro.56.012302.160938

[B34] McClure R. Balasubramanian D. Sun Y. Bobrovskyy M. Sumby P. Genco C. A. (2013). Computational analysis of bacterial RNA-Seq data. *Nucleic Acids Res.* 41:e140. 10.1093/nar/gkt444 23716638 PMC3737546

[B35] Mirzaei M. Soltani N. Sarhadi E. Pascovici D. Keighley T. Salekdeh G. H. (2012). Shotgun proteomic analysis of long-distance drought signaling in rice roots. *J. Proteome Res.* 11 348–358. 10.1021/pr2008779 22047206

[B36] Mulcahy H. Charron-Mazenod L. Lewenza S. (2010). *Pseudomonas aeruginosa* produces an extracellular deoxyribonuclease that is required for utilization of DNA as a nutrient source. *Environ. Microbiol.* 12 1621–1629. 10.1111/j.1462-2920.2010.02208.x 20370819

[B37] Neilson K. A. George I. S. Emery S. J. Muralidharan S. Mirzaei M. Haynes P. A. (2014). Analysis of rice proteins using SDS-PAGE shotgun proteomics. *Methods Mol. Biol.* 1072 289–302. 10.1007/978-1-62703-631-3_21 24136530

[B38] Oh J. Li X.-H. Kim S.-K. Lee J.-H. (2017). Post-secretional activation of protease IV by quorum sensing in *Pseudomonas aeruginosa*. *Sci. Rep.* 7:4416. 10.1038/s41598-017-03733-6 28667333 PMC5493658

[B39] Park S.-J. Kim S.-K. So Y.-I. Park H.-Y. Li X.-H. Yeom D. H. (2014). Protease IV, a quorum sensing-dependent protease of *Pseudomonas aeruginosa* modulates insect innate immunity. *Mol. Microbiol.* 94 1298–1314. 10.1111/mmi.12830 25315216

[B40] Pearson J. P. Pesci E. C. Iglewski B. H. (1997). Roles of *Pseudomonas aeruginosa* las and rhl quorum-sensing systems in control of elastase and rhamnolipid biosynthesis genes. *J. Bacteriol.* 179 5756–5767. 9294432 10.1128/jb.179.18.5756-5767.1997 PMC179464

[B41] Penesyan A. Kumar S. S. Kamath K. Shathili A. M. Venkatakrishnan V. Krisp C. (2015). Genetically and phenotypically distinct *Pseudomonas aeruginosa* cystic fibrosis isolates share a core proteomic signature. *PLoS One* 10:e0138527. 10.1371/journal.pone.0138527 26431321 PMC4592193

[B42] Perkins D. N. Pappin D. J. C. Creasy D. M. Cottrell J. S. (1999). Probability-based protein identification by searching sequence databases using mass spectrometry data. *Electrophoresis* 20 3551–3567. 10612281 10.1002/(SICI)1522-2683(19991201)20:18<3551::AID-ELPS3551>3.0.CO;2-2

[B43] Petrova O. E. Sauer K. (2009). A novel signaling network essential for regulating *Pseudomonas aeruginosa* biofilm development. *PLoS Pathog.* 5:e1000668. 10.1371/journal.ppat.1000668 19936057 PMC2774163

[B44] Pinchuk G. E. Ammons C. Culley D. E. Li S.-M. W. McLean J. S. Romine M. F. (2008). Utilization of DNA as a sole source of phosphorus, carbon, and energy by *Shewanella* spp.: ecological and physiological implications for dissimilatory metal reduction. *Appl. Environ. Microbiol.* 74 1198–1208. 18156329 10.1128/AEM.02026-07 PMC2258558

[B45] Rampioni G. Falcone M. Heeb S. Frangipani E. Fletcher M. P. Dubern J.-F. (2016). Unravelling the genome-wide contributions of specific 2-alkyl-4-quinolones and PqsE to quorum sensing in *Pseudomonas aeruginosa*. *PLoS Pathog.* 12:e1006029. 10.1371/journal.ppat.1006029 27851827 PMC5112799

[B46] Reis R. S. Pereira A. G. Neves B. C. Freire D. M. G. (2011). Gene regulation of rhamnolipid production in *Pseudomonas aeruginosa* –a review. *Bioresour. Technol.* 102 6377–6384. 10.1016/j.biortech.2011.03.074 21498076

[B47] Robinson C. (2013). *Utilization of Mucin Sulfate by Pseudomonas aeruginosa*-Importance for Cystic Fibrosis. Ph.D thesis Manchester: University of Manchester.

[B48] Sadikot R. T. Blackwell T. S. Christman J. W. Prince A. S. (2005). Pathogen–host interactions in *Pseudomonas aeruginosa* pneumonia. *Am. J. Respir. Crit. Care Med.* 171 1209–1223. 15695491 10.1164/rccm.200408-1044SO PMC2718459

[B49] Smith D. J. (2015). *Investigating the Host and Bacterial Factors in Cystic Fibrosis that Promote Persistence of Infection in the Lung.* Ph.D Thesis Brisbane: The University of Queensland.

[B50] Sonnleitner E. Valentini M. Wenner N. Haichar F. Z. Haas D. Lapouge K. (2012). novel targets of the CbrAB/Crc carbon catabolite control system revealed by transcript abundance in *Pseudomonas aeruginosa*. *PLoS One* 7:e44637. 10.1371/journal.pone.0044637 23115619 PMC3480352

[B51] Stover C. K. Pham X. Q. Erwin A. L. Mizoguchi S. D. Warrener P. Hickey M. J. (2000). Complete genome sequence of *Pseudomonas aeruginosa* PAO1, an opportunistic pathogen. *Nature* 406 959–964. 10984043 10.1038/35023079

[B52] Thaysen-Andersen M. Venkatakrishnan V. Loke I. Laurini C. Diestel S. Parker B. L. (2015). Human neutrophils secrete bioactive paucimannosidic proteins from azurophilic granules into pathogen-infected sputum. *J. Biol. Chem.* 290 8789–8802. 10.1074/jbc.M114.631622 25645918 PMC4423670

[B53] Tjaden B. (2015). *De novo* assembly of bacterial transcriptomes from RNA-seq data. *Genome Biol.* 16:1. 10.1186/s13059-014-0572-2 25583448 PMC4316799

[B54] Tralau T. Vuilleumier S. Thibault C. Campbell B. J. Hart C. A. Kertesz M. A. (2007). Transcriptomic analysis of the sulfate starvation response of *Pseudomonas aeruginosa*. *J. Bacteriol.* 189 6743–6750. 17675390 10.1128/JB.00889-07 PMC2045191

[B55] Turnbull L. Toyofuku M. Hynen A. L. Kurosawa M. Pessi G. Petty N. K. (2016). Explosive cell lysis as a mechanism for the biogenesis of bacterial membrane vesicles and biofilms. *Nat. Commun.* 7:11220. 10.1038/ncomms11220 27075392 PMC4834629

[B56] van der Ploeg J. R. Weiss M. A. Saller E. Nashimoto H. Saito N. Kertesz M. A. (1996). Identification of sulfate starvation-regulated genes in *Escherichia coli*: a gene cluster involved in the utilization of taurine as a sulfate source. *J. Bacteriol.* 178 5438–5446. 8808933 10.1128/jb.178.18.5438-5446.1996 PMC178364

[B57] Vankeerberghen A. Cuppens H. Cassiman J.-J. (2002). The cystic fibrosis transmembrane conductance regulator: an intriguing protein with pleiotropic functions. *J. Cyst. Fibros.* 1 13–29. 15463806 10.1016/s1569-1993(01)00003-0

[B58] Vizcaíno J. A. Deutsch E. W. Wang R. Csordas A. Reisinger F. Rios D. (2014). ProteomeXchange provides globally coordinated proteomics data submission and dissemination. *Nat. Biotechnol.* 32 223–226. 24727771 10.1038/nbt.2839 PMC3986813

[B59] Wagner V. E. Iglewski B. H. (2008). P. aeruginosa biofilms in CF Infection. *Clin. Rev. Allergy Immunol.* 35 124–134. 10.1007/s12016-008-8079-9 18509765

[B60] Wang G. Huang X. Li S. Huang J. Wei X. Li Y. (2012). The RNA Chaperone Hfq regulates antibiotic biosynthesis in the rhizobacterium *Pseudomonas aeruginosa* M18. *J. Bacteriol.* 194 2443–2457. 10.1128/JB.00029-12 22427627 PMC3347214

[B61] Wessel D. Flugge U. I. (1984). A method for the quantitative recovery of protein in dilute solution in the presence of detergents and lipids. *Anal. Biochem.* 138 141–143. 6731838 10.1016/0003-2697(84)90782-6

[B62] Whiteley M. Lee K. M. Greenberg E. P. (1999). Identification of genes controlled by quorum sensing in *Pseudomonas aeruginosa*. *Proc. Natl. Acad. Sci. U.S.A.* 96 13904–13909. 10570171 10.1073/pnas.96.24.13904 PMC24163

[B63] Wiegand I. Hilpert K. Hancock R. E. (2008). Agar and broth dilution methods to determine the minimal inhibitory concentration (MIC) of antimicrobial substances. *Nat. Protoc.* 3 163–175. 10.1038/nprot.2007.521 18274517

[B64] Wilson R. Sykes D. A. Watson D. Rutman A. Taylor G. W. Cole P. J. (1988). Measurement of *Pseudomonas aeruginosa* phenazine pigments in sputum and assessment of their contribution to sputum sol toxicity for respiratory epithelium. *Infect. Immun.* 56 2515–2517. 3137173 10.1128/iai.56.9.2515-2517.1988 PMC259599

[B65] Winsor G. L. Griffiths E. J. Lo R. Dhillon B. K. Shay J. A. Brinkman F. S. (2016). Enhanced annotations and features for comparing thousands of *Pseudomonas* genomes in the *Pseudomonas* genome database. *Nucleic Acids Res.* 44 D646–D653. 10.1093/nar/gkv1227 26578582 PMC4702867

[B66] Winstanley C. O’Brien S. Brockhurst M. A. (2016). *Pseudomonas aeruginosa* evolutionary adaptation and diversification in cystic fibrosis chronic lung infections. *Trends Microbiol.* 24 327–337. 10.1016/j.tim.2016.01.008 26946977 PMC4854172

[B67] Yeung A. T. Y. Janot L. Pena O. M. Neidig A. Kukavica-Ibrulj I. Hilchie A. (2014). Requirement of the *Pseudomonas aeruginosa* CbrA sensor kinase for full virulence in a murine acute lung infection model. *Infect. Immun.* 82 1256–1267. 10.1128/IAI.01527-13 24379284 PMC3957987

[B68] Zaoui C. Overhage J. Lons D. Zimmermann A. Musken M. Bielecki P. (2012). An orphan sensor kinase controls quinolone signal production via MexT in *Pseudomonas aeruginosa*. *Mol. Microbiol.* 83 536–547. 10.1111/j.1365-2958.2011.07947.x 22168309

[B69] Zhang Y. Morar M. Ealick S. E. (2008). Structural biology of the purine biosynthetic pathway. *Cell Mol. Life Sci.* 65 3699–3724. 18712276 10.1007/s00018-008-8295-8 PMC2596281

