# Supplementary material for: Catabolism of Nucleic Acids by a Cystic Fibrosis Pseudomonas aeruginosa Isolate: An Adaptive Pathway to Cystic Fibrosis Sputum Environment
Source: Front Microbiol. 2019 May 31;10:1199. doi: 10.3389/fmicb.2019.01199 (PMC6555301; doi:10.3389/fmicb.2019.01199)
Supplement: Supplementary file 2 [file Table_2.DOCX]

**TABLE ST2** SNP mutations in *P. aeruginosa* PASS4 mutants

| Mutant | PASS4 Wild-type | PASS4 Mutant | Position (codon) | Gene Identification | Gene | Product |
| --- | --- | --- | --- | --- | --- | --- |
| 1 | G | T | 354 | AOA76_RS03255 | *purK* | Phosphoribosylaminoimidazole carboxylase |
| 1 | G | A | 584 | AOA76_RS29575 | *pctA/pctC* | Chemotaxis transducer |
| 2 | G | A | 214 | AOA76_RS03255 | *purK* | Phosphoribosylaminoimidazole carboxylase |
| 3 | G | A | 214 | AOA76_RS03255 | *purK* | Phosphoribosylaminoimidazole carboxylase |
| 4 | G | T | 354 | AOA76_RS03255 | *purK* | Phosphoribosylaminoimidazole carboxylase |
| 5 | G | T | 354 | AOA76_RS03255 | *purK* | Phosphoribosylaminoimidazole carboxylase |
| 6 | G | T | 354 | AOA76_RS03255 | *purK* | Phosphoribosylaminoimidazole carboxylase |
| 7 | G | A | 1279/ 1853 | PA1874 |  | Hypothetical protein |
| 7 | G | T | 354 | AOA76_RS03255 | *purK* | Phosphoribosylaminoimidazole carboxylase |
| 8 | G | T | 354 | AOA76_RS03255 | *purK* | Phosphoribosylaminoimidazole carboxylase |
