# Supplementary material for: Catabolism of Nucleic Acids by a Cystic Fibrosis Pseudomonas aeruginosa Isolate: An Adaptive Pathway to Cystic Fibrosis Sputum Environment
Source: Front Microbiol. 2019 May 31;10:1199. doi: 10.3389/fmicb.2019.01199 (PMC6555301; doi:10.3389/fmicb.2019.01199)
Supplement: Supplementary file 3 [file Data_Sheet_1.docx]

**
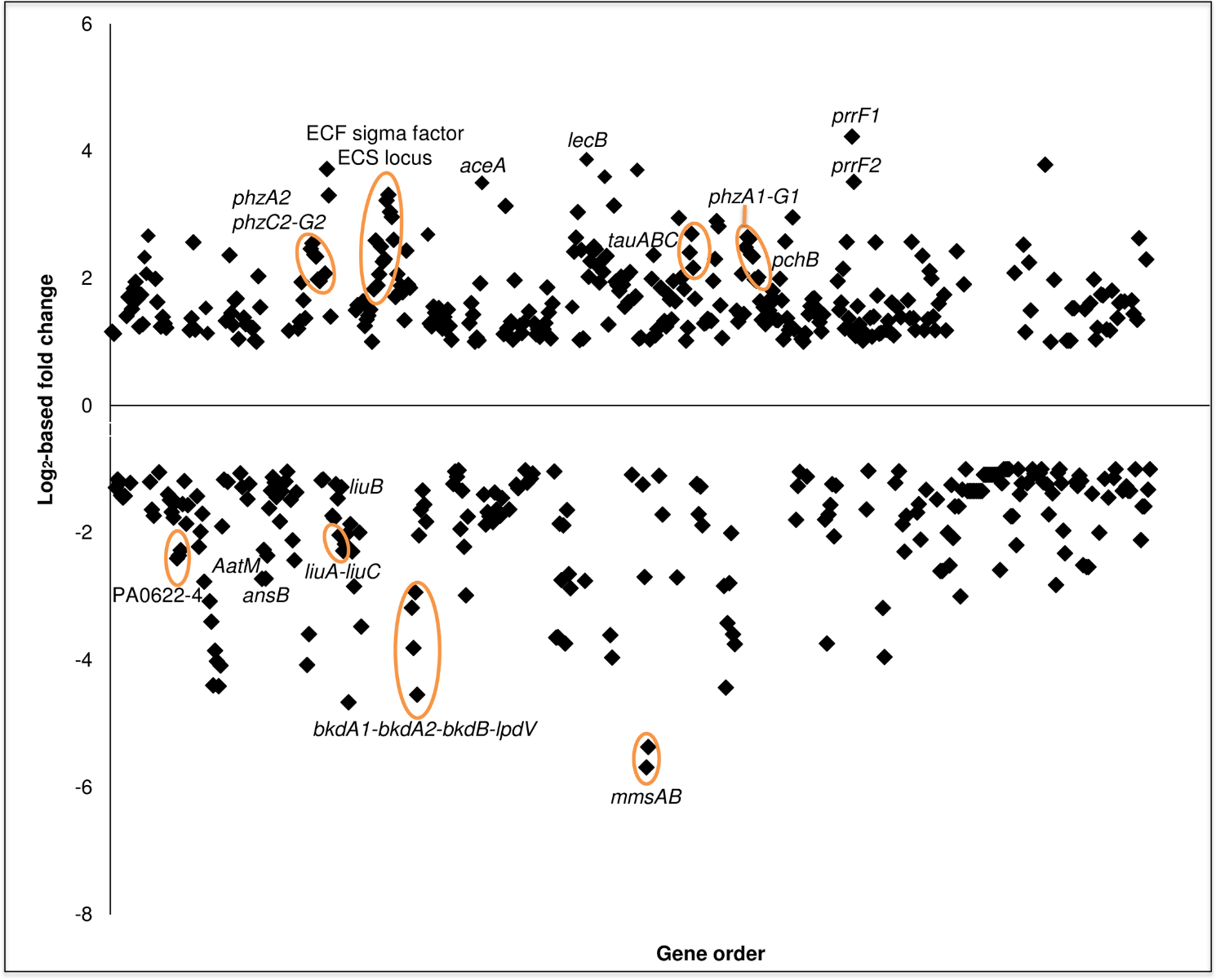
FIG S1** Differential gene expression in *P. aeruginosa* PASS4 cells grown in DNA compared to cells grown in asparagine. Each dot represents a gene within the *P. aeruginosa* PASS4 genome (x-axis) and its fold-change (log_2_) expression in DNA. Only significantly differentially expressed genes are shown (*P* value < 0.01, log_2_ fold-change 1< to < -1).

**
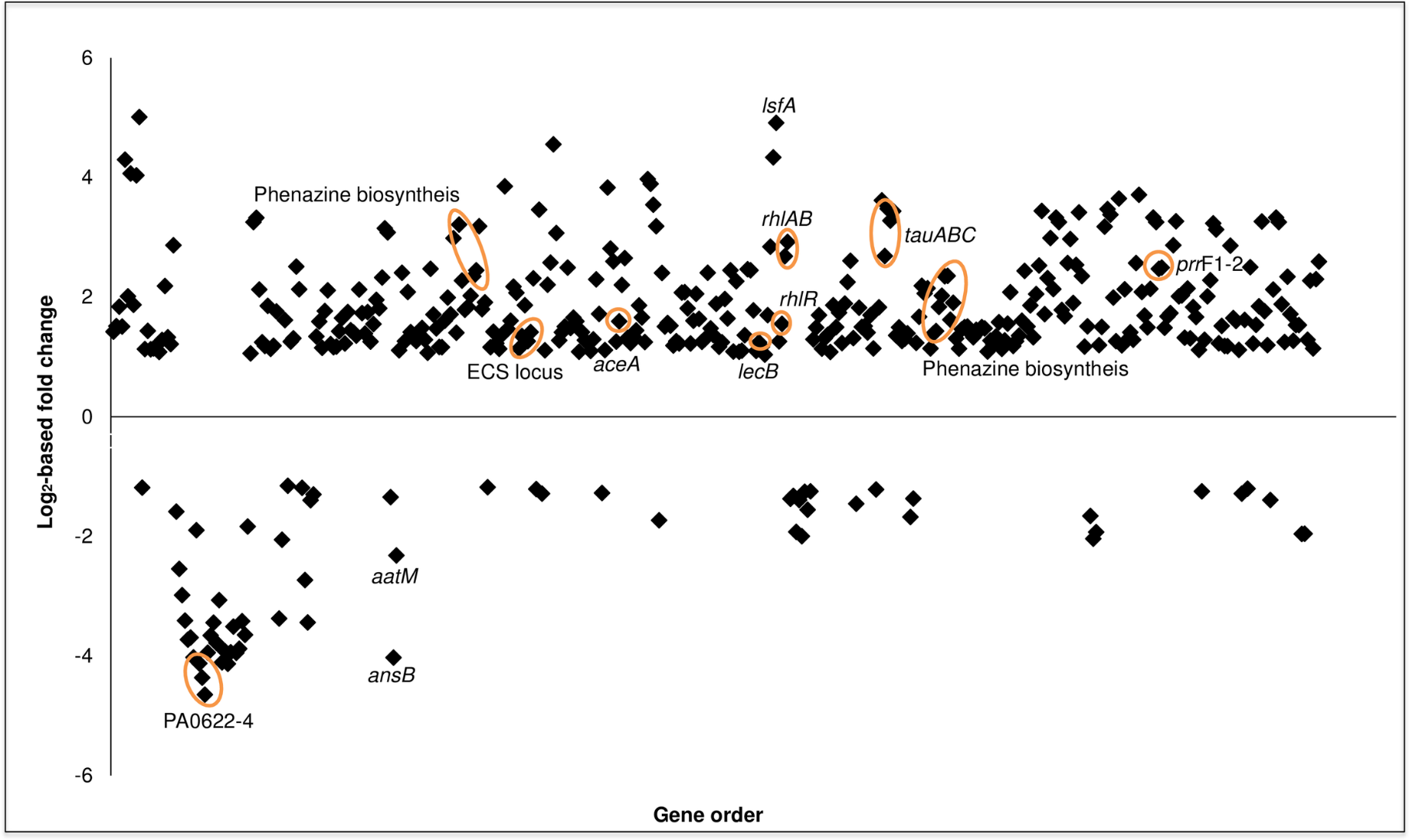
**

**FIG S2** Differential gene expression in *P. aeruginosa* PAO1 cells grown in DNA compared to cells grown in asparagine. Each dot represents a gene within the *P. aeruginosa* PAO1 genome (x-axis) and its fold-change (log_2_ scale on the y-axis) expression in DNA. Only significantly differentially expressed genes are shown *(P* value *<* 0.01, log_2_ fold-change 1< to < -1).

**
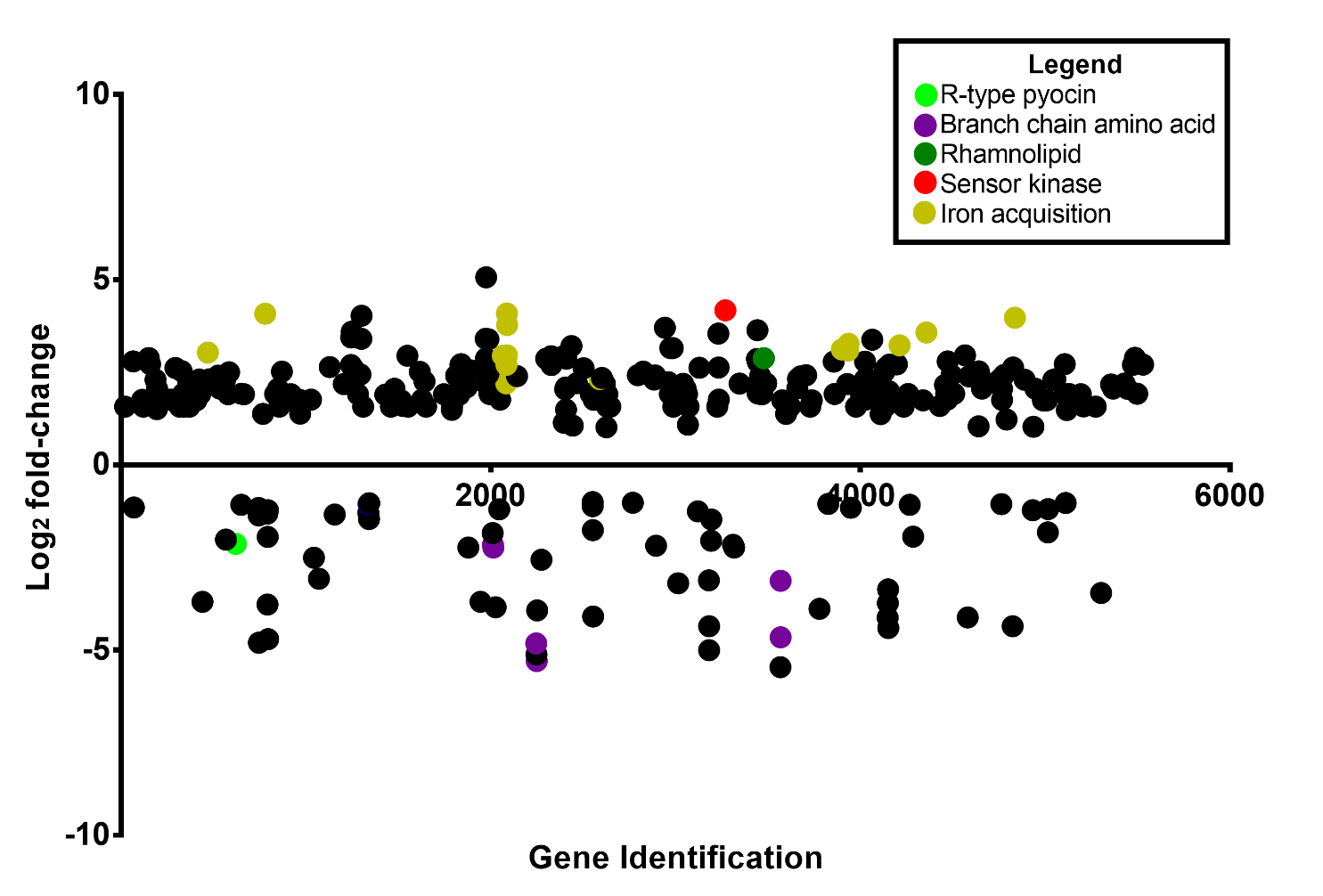
**

**FIG S3** Protein expression in *P. aeruginosa* PASS4 cells grown in DNA compared to cells grown in asparagine. Each dot represents a protein within the *P. aeruginosa* PASS4 genome (x-axis) and its fold-change (log_2_ scale on the y-axis) expression in DNA. Only significantly differentially expressed genes are shown *(P* value *<* 0.05, log_2_ fold-change 1< to < -1).


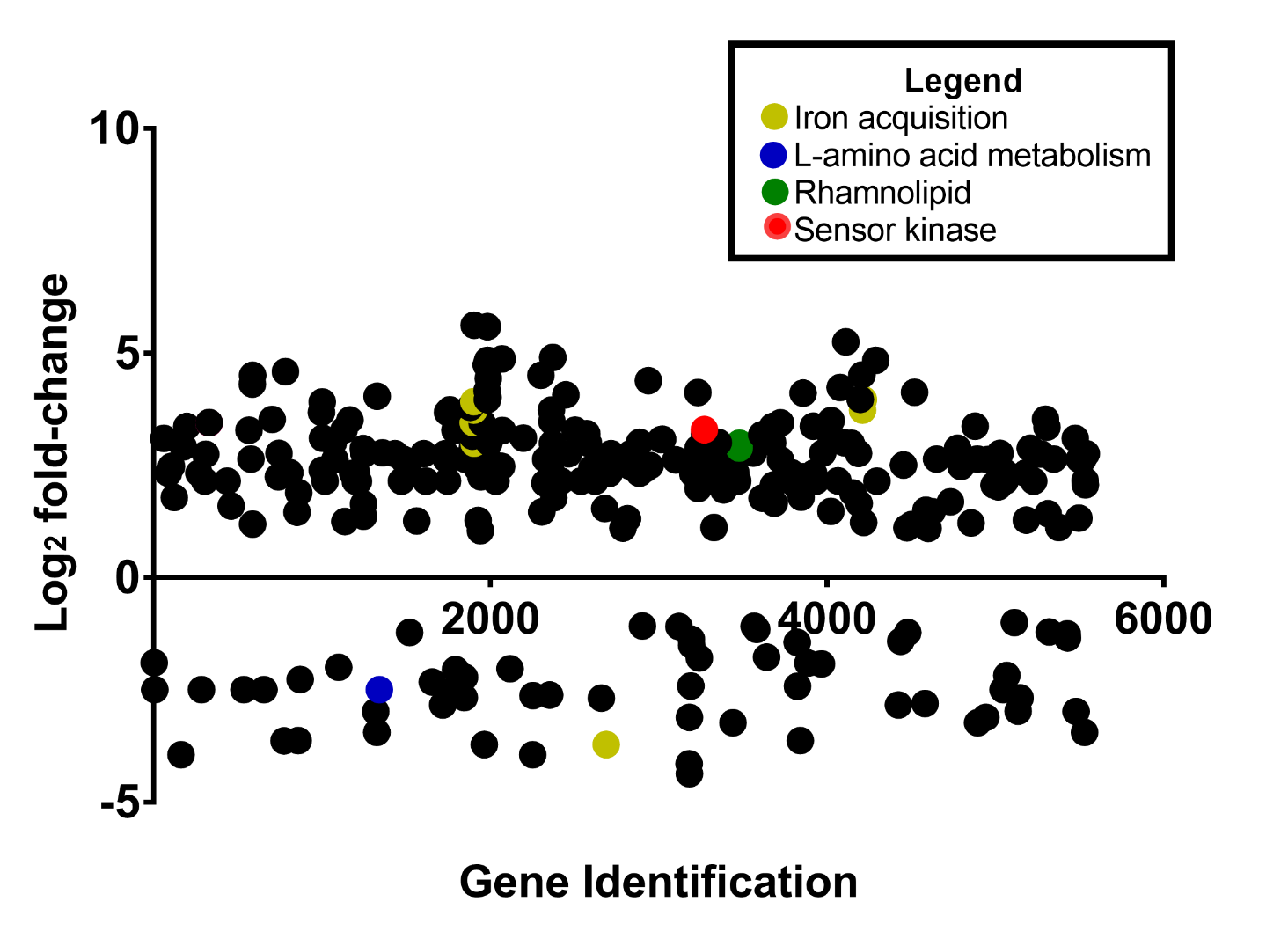


**FIG S4** Protein expression in *P. aeruginosa* PAO1 cells grown in DNA compared to cells grown in asparagine. Each dot represents a protein within the *P. aeruginosa* PAO1 genome (x-axis) and its fold-change (log_2_ scale on the y-axis) expression in DNA. Only significantly differentially expressed genes are shown *(P* value *<* 0.05, log_2_ fold-change 1< to < -1).


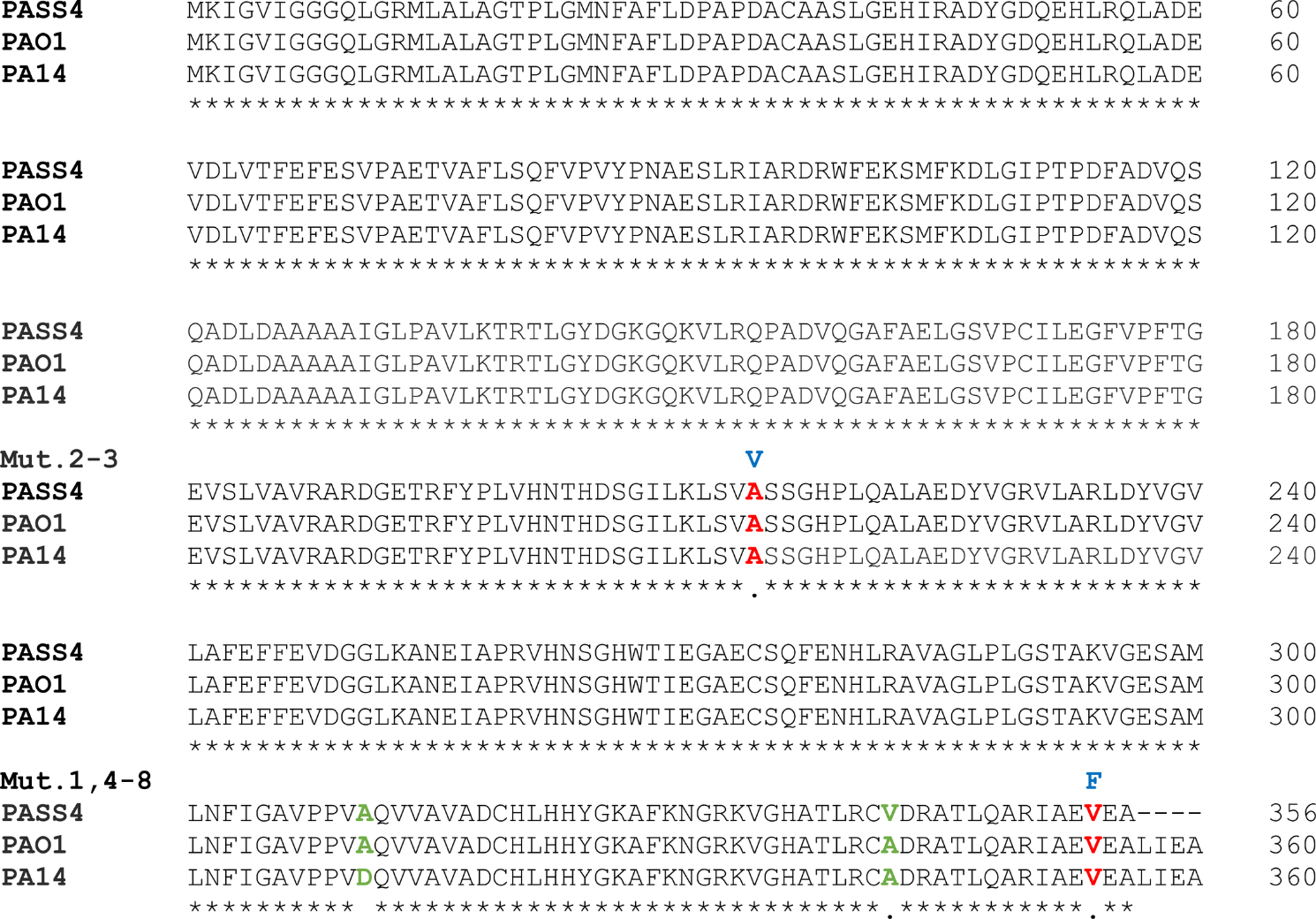


**FIG S5** Amino acid sequence alignment of the predicted amino acid sequences from the *purK* gene in *P. aeruginosa* PASS4, PAO1 and PA14. Non-identical residues are coloured green. The location of altered amino acid residues for the PASS4 mutants selected for growth on N-Acetyl-D-Glucosamine are shown above the alignment in blue, with the corresponding residue in the alignment highlighted in red.

**
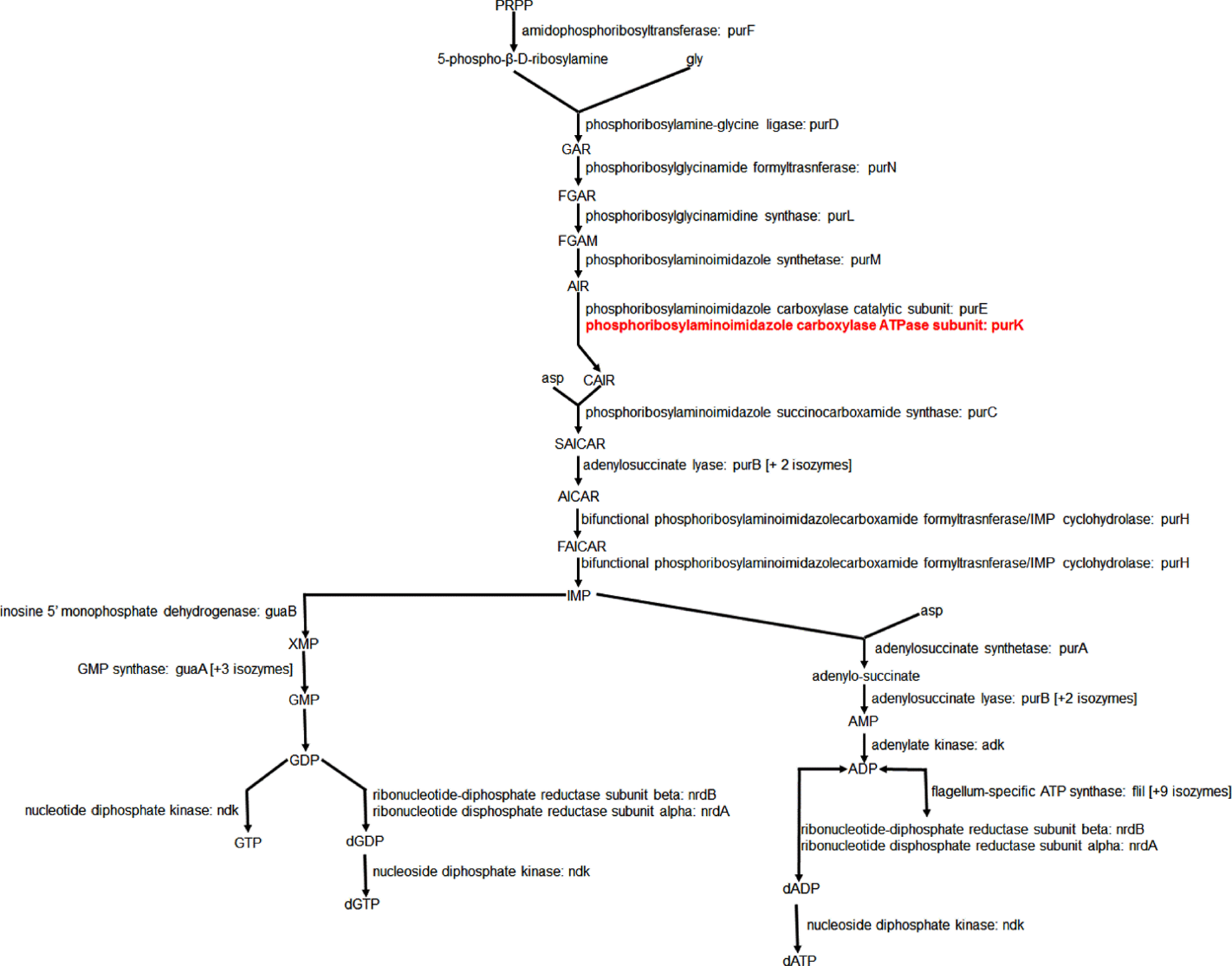
FIG S6** Graphic overview of the super-pathway of the *de novo* purine biosynthetic pathway in *P. aeruginosa*. The PASS4 mutants selected for growth on N-Acetyl-D-Glucosamine all possessed mutations in the *purK* gene, which together with its protein product are highlighted in red in this figure
